# ARMCX Family Gene Expression Analysis and Potential Prognostic Biomarkers for Prediction of Clinical Outcome in Patients with Gastric Carcinoma

**DOI:** 10.1155/2020/3575038

**Published:** 2020-06-30

**Authors:** TingAn Wang, HuaGe Zhong, YuZhou Qin, WeiYuan Wei, Zhao Li, MingWei Huang, XiaoLing Luo

**Affiliations:** ^1^Department of Gastrointestinal Surgery, Guangxi Medical University Cancer Hospital, Guangxi Clinical Research Center for Colorectal Cancer, Nanning 530021, Guangxi Zhuang Autonomous Region, China; ^2^Guangxi Medical University Cancer Hospital, Nanning 530021, Guangxi Zhuang Autonomous Region, China

## Abstract

Armadillo gene subfamily members (ARMCX1-6) are well-known to regulate protein-protein interaction involved in nuclear transport, cellular connection, and transcription activation. Moreover, ARMCX signals on cell pathways also implicated in carcinogenesis and tumor progression. However, little is known about the associations of the ARMCX subfamily members with gastric carcinoma. This study investigated the prognostic value of ARMCX subfamily mRNA expression levels with the prognosis of gastric carcinoma (GC). We retrieved the data of a total of 351 GC patients from TCGA database. Survival and gene set enrichment analyses were employed to explore the predictive value and underlying mechanism of ARMCX genes in GC. The multivariate survival analysis revealed that individually low expressions of ARMCX1 (adjusted *P* = 0.006, HR = 0.620, CI = 0.440 − 0.874) and ARMCX2 (adjusted *P* = 0.005, HR = 0.610, 95%CI = 0.432–0.861) were related to preferable overall survival (OS). The joint-effects analysis shown that combinations of low level expression of ARMCX1 and ARMCX2 were correlated with favorable OS (adjusted P = 0.003, HR = 0.563, 95%CI = 0.384–0.825). ARMCX1 and ARMCX2 were implicated in WNT and NF-kappaB pathways, and biological processes including cell cycle, apoptosis, RNA modification, DNA replication, and damage response. Our results suggest that mRNA expression levels of ARMCX subfamily are potential prognostic markers of GC.

## 1. Introduction

Gastric carcinoma, one common type of malignant tumors, is the fifth highest incidence and the second highest mortality after lung cancer worldwide [1]. Each year, more than 300,000 newly diagnosed cases and about 260,000 people die in China. The poor prognosis is due to a high incidence of advanced disease, high recurrence rate, high metastasis, and abnormal gene expression. In addition, despite great advances in the surgery and chemotherapy technology, the death rate remains high [2]. Therefore, new strategies to improve diagnosis and prognosis of gastric cancer are shortly needed.

The armadillo genes are clustered on the X chromosome, also known as X-linked (ARMCX or ALEX). In 1989, it was first discovered in the segment polarity gene armadillo in Drosophila [3, 4]. Since then, more and more related proteins have been identified and classified as armadillo repeat family. The common feature of these proteins is an amino acid sequence (arm repeats) approximately 42 residues, identified as 6-13 repeat units in all members of the family [5, 6], and each repeat domain consists of three helices, designated as H1, H2, and H3 [7–9].

The armadillo domain protein has the functions of cell contact and cytoskeletal-related protein and signal transmission by producing and transmitting signals that affect gene expression [5, 9]. Studies have revealed that armadillo repeat proteins regulate protein interactions through multiple binding domains such as nuclear transport, transcriptional activation, and cell connectivity [10]. For example, bioinformatics analysis shows that ARMCX1, ARMCX2, and ARMCX3 are encoded by an single exon, containing some ARM repeat domains, a DUF634 (domain 634 function unknown) and an N-terminal transmembrane domain [11–13].

Recent studies have shown a strong implication of different members of the Armcx1-6/Armc10 family in human tumorigenesis [14–16]. For instance, some members of the Armcx cluster can be regulated through the WNT signaling pathway by interacting with transcription factors of the E-cadherin and T cytokine/lymphoid enhancement factor (TCF/LEF) families [17, 18], which is also implicated in carcinogenesis and tumor progression [19–21].

Although the ARMCX family plays an important role in many biological processes including cell adhesion, tumorigenesis, and embryogenesis [22]. However, the relationship between ARMCX genes and gastric cancer is poorly understood. Therefore, in this study, we determined the associations between expression levels of ARMCX genes and clinical outcomes of GC prognosis, with the aim of providing insightful information regarding ARMCX genes as a novel prognostic biomarker for GC patients.

## 2. Material and Methods

### 2.1. Data Source and Patient Information

First, we identified the genes differentially expressed between normal gastric tissue and primary tumors of the ARMCX family using an online database (http://merav.wi.mit.edu/; accessed Sept 25, 2019). Then, we obtained mRNA expression levels of ARMCX1, ARMCX2, ARMCX3, ARMCX4, ARMCX5, and ARMCX6 by using The Cancer Genome Atlas (TCGA, http://tcga-data.nci.nih.gov/tcga) and OncoLnc website (http://www.oncolnc.org/; accessed Sept 25, 2019) [23].

We downloaded the clinical information of 415 gastric cancer patients from UCSC Xena (http://xena.ucsc.edu/, accessed Sept 25, 2019), including age, gender, tumor stage, survival time, and survival status. Next, a total of 351 cases were included for follow-up analysis after excluding the cases with missing medical data and 0-day survival time.

### 2.2. Characteristics of Gene Expression Levels

The high-expression and low-expression groups of ARMCX genes were distinguished according to the median of each gene. The relative expression levels of ARMCX genes in multiple normal tissues were determined with the Genotype-Tissue Expression Portal (http://www.gtexportal.org/home/, accessed Sept 25, 2019) [24].The analysis of ARMCX mRNA expression between primary gastric cancer tissue and adjacent normal tissue was done by Gene Expression Profiling Interactive Analysis (GEPIA, http://gepia.cancer-pku.cn/, accessed Sept 25, 2019) [25].

### 2.3. Bioinformatics Characteristic of ARMCX Genes

Gene function enrichment analysis of ARMCX genes was performed to disclose the biological processes and signal pathways using the Database for Annotation and Enrichment KOBAS 3.0 (http://kobas.cbi.pku.edu.cn/index.php). The analysis included biological processes and molecular function, but no results for the ARMCX family were obtained. GeneMANIA was employed to reveal the gene-gene and protein-protein interactions of ARMCX family (http://www.genemania.org/, accessed Sept 26, 2019) [26, 27]. Additionally, the relationship among ARMCX1, ARMCX2, ARMCX3, ARMCX4, ARMCX5, and ARMCX6 was evaluated using Pearson's correlation coefficient. Results with a *P* value < 0.001 were considered to be statistically significant.

### 2.4. Survival Analysis

According to the database, the 351 GC patients were, respectively, divided into low- and high-expression groups for survival analysis. Overall survival (OS) and median survival time (MST) were used to assess the prognosis of patients with gastric cancer, to evaluate the correlation of ARMCX member mRNAs with patient survival by Kaplan-Meier estimator with a log-rank test. The relative risk of survival in gastric cancer patients was assessed by calculating the hazard ratio (HR) and 95% confidence interval (CI).

### 2.5. Joint-Effects Analysis

By analyzing the TCGA data, the results have shown that only ARMCX1 and ARMCX2 had statistical significance. The combination of ARMCX1 and ARMCX2 was investigated by joint-effects analysis. The combination included group 1 (low ARMCX1 and low ARMCX2 expression), group 2 (low ARMCX1 and high ARMCX2 expression, High ARMCX1 and low ARMCX2 expression), and group 3 (high ARMCX1 and high ARMCX2 expression). In addition, according to the results of TCGA database, age and tumor stage were adjusted in the Cox proportional hazards regression model.

### 2.6. Nomogram Model

Due to the clinical characteristics and risk score, a nomogram prediction model was constructed to evaluate the individual prognosis. Furthermore, the probable utility of the ARMCX family in predicting clinical grade was evaluated. In terms of clinical data and survival analysis, age, tumor stage, and ARMCX expression level were included in the risk model after Cox proportional risk regression model adjustment. Scores for each factor could be counted, and 1-year, 5-year, and 10-year survival rates also can be calculated [28].

### 2.7. Gene Set Enrichment Analysis

In order to explore the difference in biological functions and pathways in the survival of GC between low- and high-ARMCX gene expression groups, the potential mechanism in the molecular signature database (MSigDB) of c2 (c2.all.v6.1. Symbols) and c5 (c5.all.v6.1. Symbols) was studied by GSEA (http://software.broadinstitute.org/gsea/index.jsp, accessed Sept 27, 2019) [29–31].The nominal *P* value < 0.05 and the false discovery rate (FDR) <0.25 for the enriched gene sets in GSEA were statistically significant.

### 2.8. Statistical Analysis

Survival analysis was carried out by Kaplan-Meier and the log-rank test to calculate MSTs and *P* values. The crude or adjusted HR and 95% CI were calculated using the Cox proportional risk regression model for univariate and multivariate survival analyses. The Benjamini Hochberg procedure was employed for multiple tests of FDR in GSEA to control [31–33], and *P* < 0.05 was considered statistically significant. GraphPad Prism v.6.0 (La Jolla, CA) was used to draw vertical scatter plots and survival curves. SPSS software v.22.0 (IBM, Chicago, IL, USA) was employed for statistical analysis.

## 3. Results

### 3.1. ARMCX mRNA Expression Analysis

**Figure 1:**
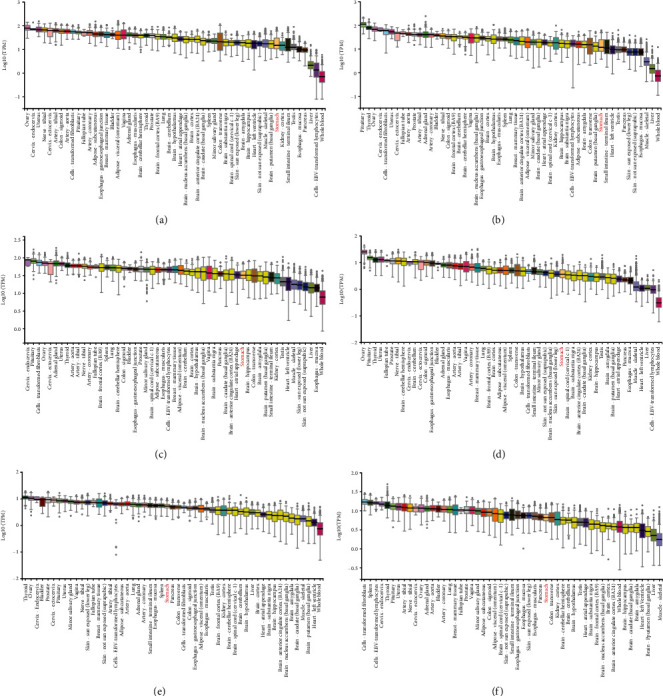
Matrix graphs of the relative expression levels of ARMCX genes in multiple normal tissues were determined with the GTEx Portal. ARMCX5 was expressed at a medium level (e), whereas the other ARMCX genes (ARMCX1, ARMCX2, ARMCX3, ARMCX4, and ARMCX6) were expressed at low levels (a-d, f).

**Figure 2:**
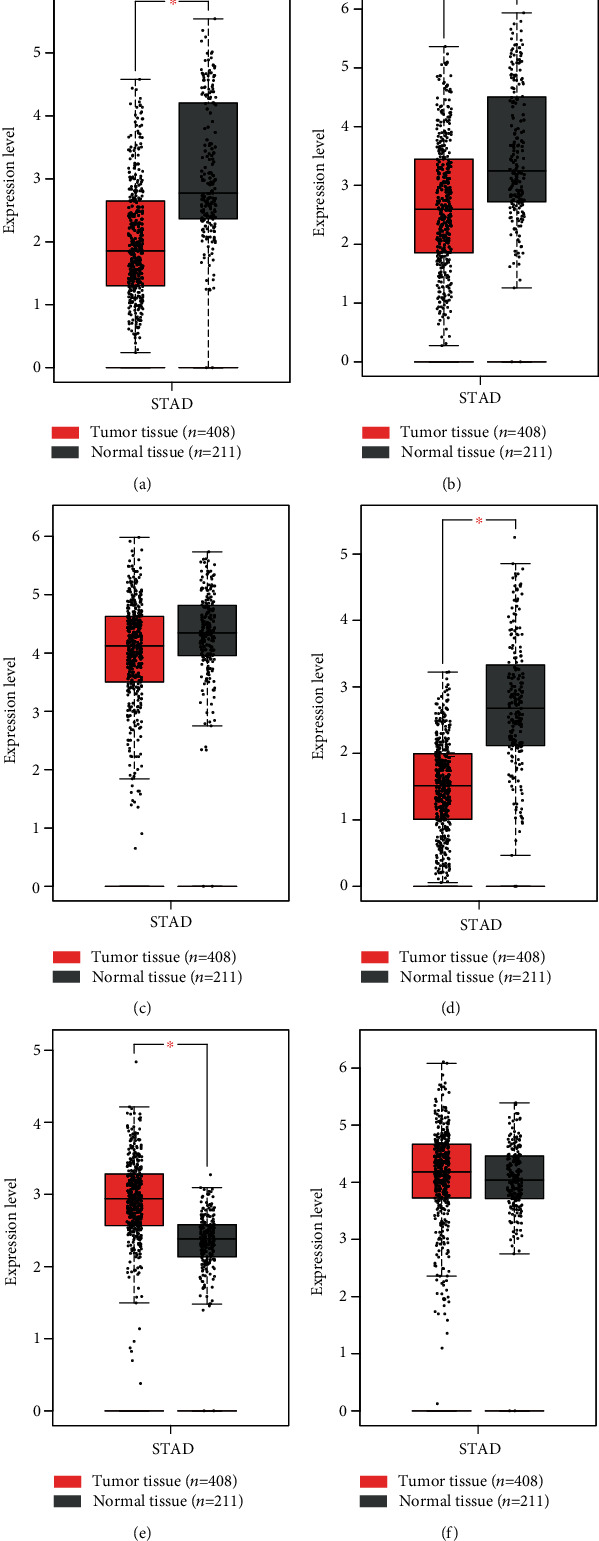
Boxplots of ARMCX family gene levels in primary gastric tumors and adjacent tissues from databases. ARMCX1, ARMCX2, and ARMCX4 were lowly expressed in primary gastric tumors (a, b, d). ARMCX5 was less expressed in normal gastric tissues (e). The expression levels of ARMCX3 and ARMCX6 have no significant difference between gastric tumors and normal gastric tissues (c, f). ^∗^*P* < 0.05.

In human normal stomach tissue, ARMCX5 was expressed at a medium level (Figure 1(e), whereas the other ARMCX genes (ARMCX1, ARMCX2, ARMCX3, ARMCX4, and ARMCX6) were expressed at low levels (Figures 1(a)-1(d) and 1(f)), compared with other normal tissues. Box plots of ARMCX1-6 genes were downloaded from GEPIA as shown in Figure 2. ARMCX1, ARMCX2, and ARMCX4 were lowly expressed in primary gastric tumors and has a high expression in normal gastric tissues. However, conversely, ARMCX5 was less expressed in normal gastric tissues than in primary gastric tumors. The expression levels of ARMCX3 and ARMCX6 have no significant difference between gastric tumors and normal gastric tissues.

### 3.2. Bioinformatics and Functional Annotation Analyses of the ARMCX Genes

**Figure 3:**
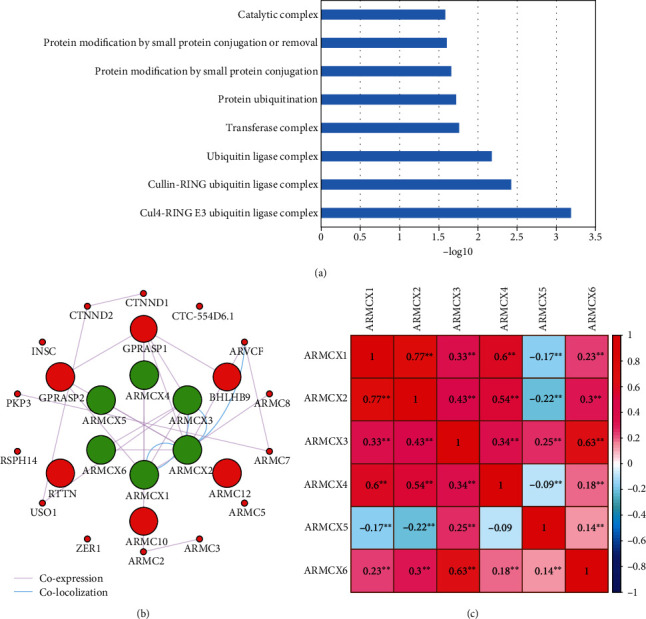
Functional assessment and bioinformatics analysis of ARMCX family genes (a). GeneMANIA constructed the gene-gene interaction network of the ARMCX family (b). Matrix graphs of Pearson's correlations of ARMCX family gene expression levels (c). ^∗∗^*P* < 0.001.

Enrichment and functional analyses by KOBAS revealed that ARMCX genes were significantly enriched in ubiquitin ligase complex and the process of protein modification (Figure 3(a)). However, we have not found any associations of the ARMCX family using Kyoto Encyclopedia of Genes and Genomes (KEGG) and Database for Annotation, Visualization, and Integrated Discovery (DAVID) analyses. By analyzing gene-gene and protein-protein interaction networks, we confirmed that the ARMCX family had strong protein homology and coexpression at both gene and protein levels, as shown in Figure 3(b).

### 3.3. Correlation Analysis Value Assessment of the ARMCX Family

Coexpression analyses of individual ARMCX genes were analyzed using Pearson's correlation coefficient. The expression level of ARMCX1, ARMCX2, ARMCX3, and ARMCX6 was correlated with each other. Furthermore, there was no significant correlation between the expressions of ARMCX4 and ARMCX5, but both the expressions of ARMCX4 and ARMCX5 all related to the other members of the ARMCX family (^∗∗^*P* < 0.01; Figure 3(c)).

### 3.4. Clinical Characteristics of GC Patients

There were 351 GC patients who had prognosis information included in the current study; UCSC Xena dataset is shown in Table 1. The univariable survival analysis revealed that age and tumor stage were correlated with MST in combination with clinical data (*P* = 0.017 and *P* < 0.001, respectively), and preliminary stage was significantly correlated with favorable MST (2197 days, *P* < 0.001, HR = 0.260, 95%CI = 0.126–0.537). On the other hand, gender was not associated with MST.

### 3.5. Survival Analysis of the ARMCX Gene Family

**Figure 4:**
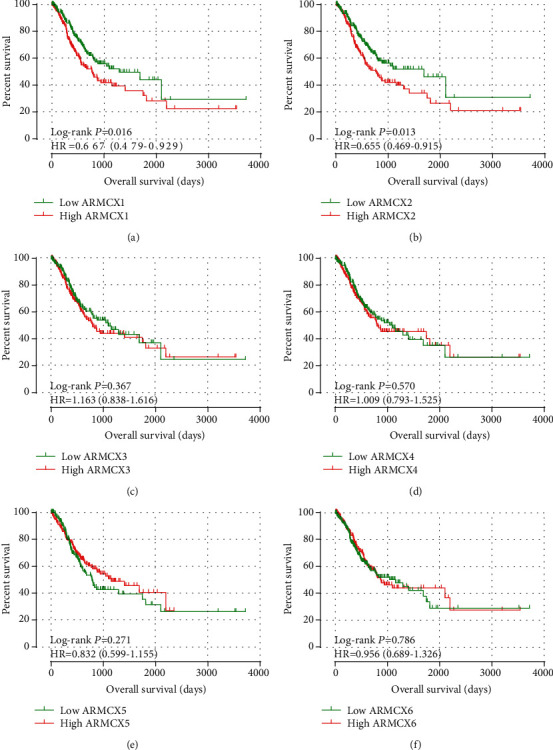
Univariate curves of OS of ARMCX family genes in GC. The lower expression levels of ARMCX1 and ARMCX2 were significantly associated with satisfactory OS results (a, b). The expression of ARMCX3, ARMCX4, ARMCX5, and ARMCX6 mRNA did not have significant prognostic value for OS (c, d).

Survival analysis is shown in Table 2 and Figure 4. Due to the age and tumor stage that were related with MST, both age and tumor stage were analyzed using the multivariate Cox proportional risk regression model. In univariate survival analysis, lower expression levels of ARMCX1 and ARMCX2 were significantly associated with satisfactory OS results (log-rank *P* = 0.016, HR = 0.667, 95%CI = 0.479–0.929; log-rank *P* = 0.013, HR = 0.655, 95%*CI* = 0.469–0.915, respectively; Figures 4(a) and 4(b)). The expression of ARMCX3, ARMCX4, ARMCX5, and ARMCX6 mRNA did not have a significant prognostic value for OS (log-rank *P* = 0.367, 0.570, 0.271, and 0.786, respectively; Figures 4(c)-4(f)).

### 3.6. Joint-Effects Analysis of ARMC1 and ARMCX2

**Figure 5:**
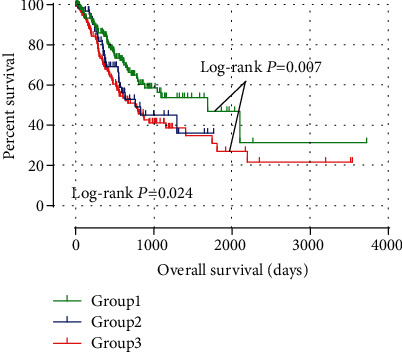
Survival curves for joint-effects analysis of the combination of ARMCX1 and ARMCX2 genes in the TCGA database. Low-expression levels of ARMCX1 and ARMCX2 in group 1 were significantly correlated with better clinical outcome. In group 3, high expression of ARMCX1 and ARMCX2 was correlated with poor OS.

Based on the findings in the multivariate survival analysis, ARMCX1 and ARMCX2 were associated with a significantly different survival. A joint-effects analysis was employed to further determine the combined effects in prognostic prediction of ARMCX1 and ARMCX2 (grouped as summarized in Table 3). The combination of ARMCX1 and ARMCX2 included group 1, group 2 and group 3, and results are shown in Table 4. Group 1 had the longest MST of 1686 days (adjusted *P* = 0.003), while group 3 had the shortest MST of 762 days (adjusted *P* = 0.012). Kaplan–Meier survival analyses of ARMCX1 and ARMCX2 are shown in Figure 5. Low expression levels of ARMCX1 and ARMCX2 in group 1 were significantly correlated with better clinical outcome. In group 3, high expression of ARMCX1 and ARMCX2 was correlated with poor OS (log-rank *P* = 0.007).

### 3.7. Nomogram Model

**Figure 6:**
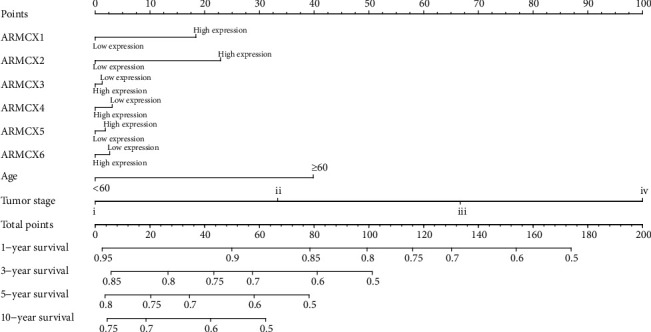
Nomogram for predicting the 1-, 3-, 5-, and 10-year events (death) with risk scores and clinical parameters. The high expression levels of ARMCX1 and ARMCX2, age of the patient (>60 years old), and advanced tumor stage established a prognostic feature that conduced to the highest risk for poor OS.

Nomogram risk scoring includes age, tumor stage, and the expression level of ARMCX1 and ARMCX2 to calculate 1-year, 5-year, and 10-year related survival rates. The higher total points, the lower survival rate, and the results substantiated that high expression levels of ARMCX1 and ARMCX2, age of the patient (>60 years old), and advanced tumor stage established a prognostic feature that conduced to the highest risk for poor OS (Figure 6).

### 3.8. Gene Set Enrichment Analysis

**Figure 7:**
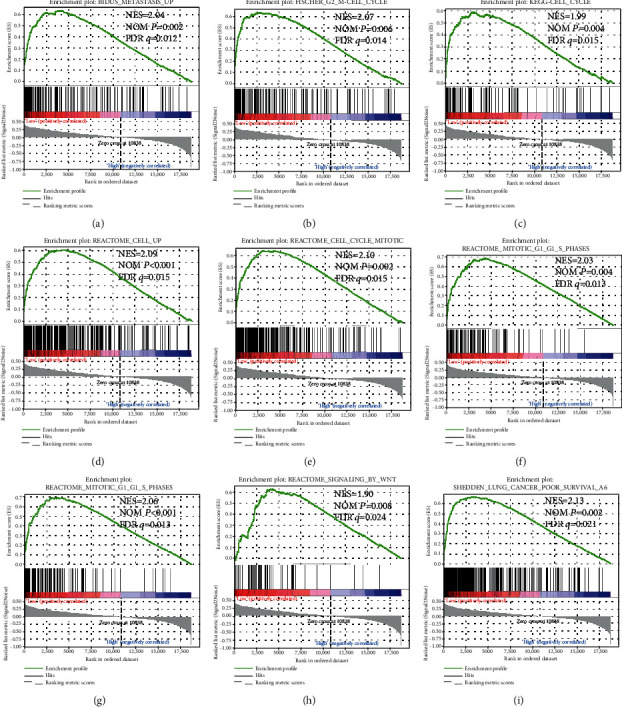
GSEA results of low *ARMCX1* expressed in GC patients, using gene set c2. The low ARMCX1 expression was involved in the WNT signaling pathway, regulation of cell metastasis (a) and cell cycle biological processes (b-h), and poor survival of lung cancer (i). Abbreviations: FDR: false discovery rate; GSEA: gene set enrichment analysis; KEGG: Kyoto Encyclopedia of Genes and Genomes; NES: normalized enrichment score; NOM: nominal.

**Figure 8:**
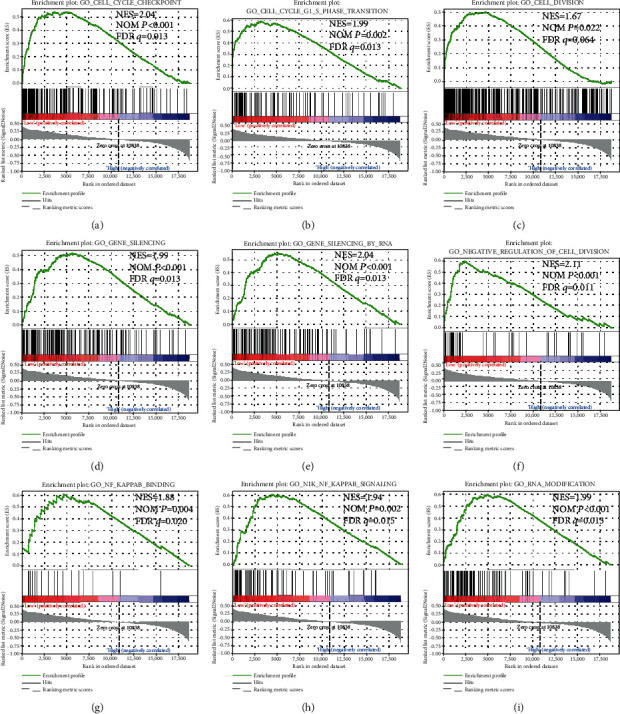
GSEA results of low *ARMCX1* expressed in GC patients, using gene set c5. The low ARMCX1 is also involved in cell division (c), cell cycle (a, b), gene silencing (d), RNA modification (i), and the NF-kappaB signaling pathway (g, h). Abbreviations: FDR: false discovery rate; GSEA: gene set enrichment analysis; KEGG: Kyoto Encyclopedia of Genes and Genomes; NES: normalized enrichment score; NOM: nominal.

**Figure 9:**
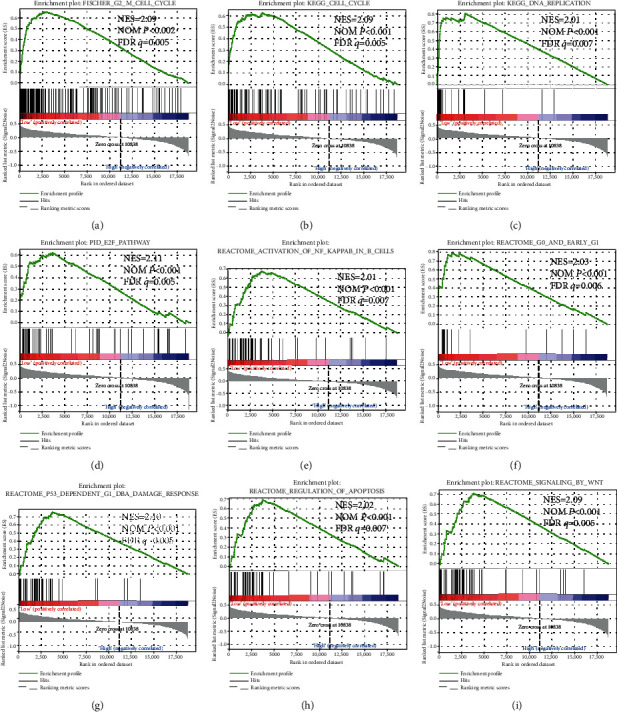
GSEA results of low *ARMCX2* expressed in GC patients, using gene set c2. The low expression of ARMCX2 was correlated to the cell cycle biological process (a, b, and f), regulation of apoptosis (h), DNA replication (c) and damage response (g), and the E2F, WNT, and NF-kappaB signaling pathways (d, i, and e). Abbreviations: FDR: false discovery rate; GSEA: gene set enrichment analysis; KEGG: Kyoto Encyclopedia of Genes and Genomes; NES: normalized enrichment score; NOM: nominal.

**Figure 10:**
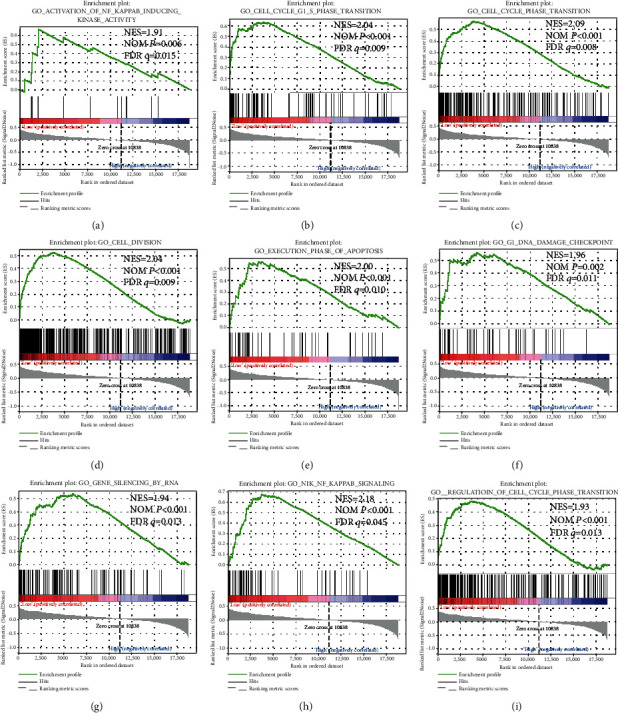
GSEA results of low ARMCX2 expressed in GC patients, using gene set c5. The low ARMCX2 expression is involved in biological process of cell division (d), cell cycle (c, i), apoptosis (e), gene silencing (g), DNA damage checkpoint (f), and the NF-kappaB signaling pathway (a). Abbreviations: FDR: false discovery rate; GSEA: gene set enrichment analysis; KEGG: Kyoto Encyclopedia of Genes and Genomes; NES: normalized enrichment score; NOM: nominal.

In order to further explore the underlying mechanisms of ARMCX genes in GC prognosis, we used the PAAD genome-wide RNA sequencing dataset for GSEA. GSEA results of the c2 reference gene set revealed that a low ARMCX1 expression was involved in the WNT signaling pathway, regulation of cell metastasis (Figure 7(a)) and cell cycle biological processes (Figures 7(b)-7(h)), and poor survival of lung cancer (Figure 7(i)). Also, the enrichment of c5 indicates that low ARMCX1 is also involved in cell division (Figure 8(c)), cell cycle (Figures 8(a) and 8(b)), gene silencing (Figure 8(d)), RNA modification (Figure 8(i)), and NF-kappaB signaling pathway (Figures 8(g) and 8(h)). GSEA results of c2 enrichments reveal that the low expression of ARMCX2 was correlated to the cell cycle biological process (Figures 9(a), 9(b), and 9(f)), regulation of apoptosis (Figure 9(h)), DNA replication (Figure 9(c)) and damage response (Figure 9(g)), and E2F, WNT, and NF-kappaB signaling pathways (Figures 9(d), 9(e), and 9(e)), whereas the c5 enrichments suggest that low ARMCX2 expression is involved in the biological process of cell division (Figure 10(d)), cell cycle (Figures 10(b), 10(c), and 10(i)), apoptosis (Figure 10(e)), gene silencing (Figure 10(g)), DNA damage checkpoint (Figure 10(f)), and the NF-kappaB signaling pathway (Figure 10(a)). Moreover, the remaining results of this study can be seen in Supplementary Tables 1 and 1.

## 4. Discussion

In our present study, we elucidated the associations between the expression levels of ARMCX 1-6 genes with the prognosis of GC patients. Our research disclosed that ARMCX 1 and ARMCX 2 contribute significantly to OS, but ARMCX 3-6 show no significant association with OS. Thus, the expression levels of ARMCX 1 and ARMCX 2 both alone and in combination may serve as potential biomarkers of GC.

In 1989, the armadillo family proteins were first discovered in the polar gene fragment of Drosophila [3]. Subsequently, more and more proteins containing arm repeats have been analyzed and sequenced. Armadillo repeats containing x-chain (ARMCX 1-6) are involved in many biological processes, such as mediating protein-protein interactions and intervening in cell assembly, nuclear transport, and transcriptional activation [34]. Many studies have demonstrated that ARMCX is associated with the risk and prognosis of several diseases. For instance, the ARMCX family plays an important role in embryogenesis and tumorigenesis [22]. Scholars have found that some members of the ARMCX protein family (Armcx1-3) were underexpressed in several cancers of epithelial origin, including the lung, prostate, colon, and pancreatic [11].

ARMCX1, ARMCX2, and ARMCX3 are located in the chromosome region xq21.33-q22.2, respectively. Their amino N-terminal region has a transmembrane domain, indicating that these proteins may be located in the membrane structure of cells. ARMCX3 has been found to be a complete membrane protein of the mitochondrial outer membrane, which functions by interacting with transcription regulator Sox10 [12]. In addition, ARMCX4, ARMCX5, and ARMCX6 were located in chromosome regions xq22.1, xq22.1-q22.3, and xq21.33-q22.3, respectively. Studies have shown that ARMCX5 can be activated by binding to the oncogene ZnF217 [35] and ARMCX6 upexpressed at least 2-fold in peripheral blood monocytes of rheumatoid arthritis patients compared to those identified using oligonucleotide array [36]. Moreover, regardless of their function in other diseases, they are associated with tumorigenesis and were initially described as presumed tumor suppressors [11].

Here, we downloaded and analyzed data from GEO online database to determine the potential relationship between ARMCX mRNA expression and clinical outcomes of patients with gastric cancer. We observed significant differences in the expression of ARMCX1, ARMCX2, ARMCX4, and ARMCX5 between primary tumors and adjacent normal tissues, without ARMCX3 and ARMCX6. More importantly, ARMCX1 and ARMCX2 are more highly expressed in adjacent normal tissues than in tumor tissues, leading to better OS in patients with gastric cancer, although the mechanism of action needs further clarification.

In addition, a comprehensive survival analysis of the current prognostic characteristics of ARMCX was performed by establish a nomogram, and stratified joint-effects survival analysis was conducted to explore its potential application. The results indicated that high ARMCX expression was an independent risk factor as a prognostic characteristic for patients with gastric cancer, and the relevant risk score could be used as a prognostic indicator. Nomogram, composed of risk score and other clinical information such as age and tumor stage, is an important prognostic risk assessment system for gastric cancer.

To explore the underlying mechanism of ARMCX genes in gastric cancer prognosis, we used a genome-wide RNA sequencing dataset in GSEA. The NF-kappaB, E2F and WNT signaling pathways, the cell cycle, and gene silencing were significantly enriched in the ARMCX1 and ARMCX2 low-expression groups.

It is well established that, as members of the armadillo (Arm) family, *β*-catenin and adenomatous polyposis coli (APC) are important components of the WNT signaling pathway. Moreover, WNT signaling plays an important role in a variety of biological processes, such as tumorigenesis, embryonic development, and stem cell maintenance [37–39]. *β*-Catenin, which is a multifunctional protein, plays an essential role in a variety of biological responses. For instance, in the WNT signaling pathway, *β*-catenin works by interact with E-cadherin and TCF/LEF transcription factors, respectively [40, 41]. APC can regulate the WNT signaling pathway by synergistically acting with casein kinases 1, glycogen synthase kinase-3b, and AXIN to induce degradation of *β*-catenin [37, 42, 43]. On the basis of GSEA results, we deduced that both ARMCX1 and ARMCX2 were involved in the pathway and biological processes that are associated with the progress and treatment of gastric cancer and may serve as a GC prognostic marker. Once these results are verified, ARMCX1 and ARMCX2 may be used as biomarkers in combination with other clinical factors to facilitate the selection of diagnosis and treatment decisions for GC and to benefit patients with better clinical outcomes.

Although significant results have been achieved in the current study, there are still some deficiencies to be considered. First, the results of this study were obtained from a single cohort in the TCGA database, and its demographic characteristics may not be representative of all patient groups. Therefore, genetic changes may have deviation and require further validation in other GC groups. Second, the clinical information from TCGA was incomplete. Therefore, we cannot conduct a comprehensive stratification analysis in the Cox proportional risk regression model including all layers. Third, the mechanism between the above ARMCX and WNT signaling pathways affecting the clinical prognosis of GC still needs to be determined.

## 5. Conclusion

Our present study has determined that ARMCX has a potential prognostic value for gastric cancer and may have clinical application value. In addition, further basic research is needed to clarify the specific mechanisms of ARMCX in GC.

## Figures and Tables

**Table 1:** Clinical data characteristic of 351 GC patients.

Items	Cases (total *n* = 351)	No. of events (%)	MST (days)	Crude *P*	Crude HR (95% CI)
*Age*					
≥60	239	108 (45.2)	766	0.017	Ref.
<60	106	35 (33.0%)	1811		0.629 (0.429-0.923)
Missing	6				
Gender					
Male	236	100 (42.4%)	869	0.184	Ref.
Female	125	44 (35.2%)	1043		0.787 (0.552-1.122)
Missing	0				
Tumor stage					
i	47	11 (23.4%)	2197	<0.001	0.260 (0.126-0.537)
ii	109	34 (31.2%)	1686		0.424 (0.247-0.728)
iii	147	69 (46.9%)	779		0.643 (0.397-1.042)
iv	35				
Missing	13	22 (62.9%)	476		Ref.

Abbreviations: MST: median survival time; Ref.: reference; HR: hazard ratio; 95% CI: 95% confidence interval.

**Table 2:** Univariate and multivariate survival analyses of the ARMCX family.

Items	Cases (total *n* = 351)	No. of events (%)	MST (days)	Crude *P*	Crude HR (95% CI)	Adjusted *P*	Adjusted HR (95% CI)
ARMCX1							
Low	175	61 (34.6%)	1294	0.016	0.667 (0.479-0.929)	0.006	0.620 (0.440-0.874)
High	176	83 (47.6%)	766		Ref.		Ref.
ARMCX2							
Low	176	58 (33.0%)	1686	0.012	0.655 (0.469-0.915)	0.005	0.610 (0.432-0.861)
High	175	86 (49.1%)	762		Ref.		Ref.
ARMCX3							
Low	176	66 (37.5%)	1095	0.366	Ref.	0.690	Ref.
High	175	78 (44.6%)	794		1.163 (0.838-1.616)		1.072 (0.763-1.506)
ARMCX4							
Lo	176	70 (39.8%)	1095	0.570	Ref.	0.220	Ref.
High	175	74 (42.3%)	801		1.099 (0.793-1.525)		1.238 (0.880-1.741)
ARMCX5							
Low	176	76 (43.2%)	728	0.271	Ref.	0.507	Ref.
High	175	68 (38.9%)	1153		0832 (0.599-1.155)		0.891 (0.634-1.253)
ARMCX6							
Low	176	73 (41.5%)	1153	0.786	Ref.	0.929	Ref.
High	175	71 (40.6%)	874		0.956 (0.689-1.326)		1.016 (0.722-1.428)

Abbreviations: MST: median survival time; Ref.: reference; HR: hazard ratio; 95% CI: 95% confidence interval; Notes: Adjusted *P*, adjustment for age and tumor stage.

**Table 3:** Grouping information for joint-effects analysis.

Group	Combinations
1	Low ARMCX1+low ARMCX2
2	Low ARMCX1+high ARMCX2 High ARMCX1+low ARMCX2
3	High ARMCX1+high ARMCX2

Abbreviations: ARMCX: arm protein lost in epithelial cancers, X chromosome.

**Table 4:** Joint-effects analysis of the combination of ARMCX1 and ARMCX2.

Items	Cases (total *n* = 351)	No. of events (%)	MST (days)	Crude *P*	Crude HR (95% CI)	Adjusted *P*	Adjusted HR (95% CI)
Group 1	143	46 (32.2%)	1686	0.007	0.600 (0.414-0.870)	0.003	0.563 (0.384-0.825)
Group 2	65	27 (41.5%)	766	0.549	0.873 (0.559-1.362)	0.506	0.854 (0.537-1.358)
Group 3	143	71 (49.7%)	762	0.025	Ref.	0.012	Ref.

Abbreviations: MST: median survival time; Ref.: reference; HR: hazard ratio; 95% CI: 95% confidence interval; Notes: Adjusted *P*, adjustment for age and tumor stage.

## Data Availability

The datasets used and/or analyzed during the current study are available from the corresponding author on reasonable request and TCGA data base.

## Supplementary Materials

Supplementary 1 Supplementary Table 1: GSEA KEGG (c2.all.v6.2.symbols.gmt) results of ARMCX1 and ARMCX2.

Supplementary 2 Supplementary Table 2: GSEA GO (c5.all.v6.2.symbols.gmt) functional analysis results of ARMCX1 and ARMCX2.
