# Corrigendum to “ARMCX Family Gene Expression Analysis and Potential Prognostic Biomarkers for Prediction of Clinical Outcome in Patients with Gastric Carcinoma”

**DOI:** 10.1155/2021/3179693

**Published:** 2021-02-09

**Authors:** TingAn Wang, HuaGe Zhong, YuZhou Qin, WeiYuan Wei, Zhao Li, MingWei Huang, XiaoLing Luo

**Affiliations:** ^1^Department of Gastrointestinal Surgery, Guangxi Medical University Cancer Hospital, Guangxi Clinical Research Center for Colorectal Cancer, Nanning, 530021 Guangxi Zhuang Autonomous Region, China; ^2^Guangxi Medical University Cancer Hospital, Nanning, 530021 Guangxi Zhuang Autonomous Region, China

In the article titled “ARMCX Family Gene Expression Analysis and Potential Prognostic Biomarkers for Prediction of Clinical Outcome in Patients with Gastric Carcinoma” [1], there are errors in both the grant number and the funder name stated in the Acknowledgements section, which should be updated as follows:

“We sincerely appreciate TCGA (https://cancergenome.nih.gov/) and UCSC Xena (http://xena.ucsc.edu/) for sharing the GC data and for Dr Liucheng Wu for his advice with the language consulting of the manuscript. The study was supported by the National Natural Science Foundation of GuangXi (No. 2019GXNSFBA185041) and National Natural Science Foundation of China (No. 81760521).”
